# Non-pharmacological interventions for people presenting in crisis to emergency departments and inpatient wards: a scoping, typology, and systematic review

**DOI:** 10.1192/j.eurpsy.2023.642

**Published:** 2023-07-19

**Authors:** J. P. Huber, A. Milton, M. Brewer, L. Norrie, N. Glozier

**Affiliations:** ^1^Psychiatry, St Vincent’s Hospital, Sydney; ^2^Faculty of Medicine and Health, The University of Sydney; ^3^ ARC Center of Excellence for Children and Families over the Life Course; ^4^Central Clinical School Faculty of Medicine and Health, The University of Sydney, Sydney, Australia

## Abstract

**Introduction:**

People presenting to hospital in a crisis of mental ill-health usually present via Emergency Departments, and are often admitted for brief interventions. Unlike drug treatments, the evidence base for brief non-pharmacological interventions has not been systematically evaluated.

**Objectives:**

1. To describe brief non-pharmacological interventions used in Emergency Departments and inpatient psychiatric units, for those in a crisis of mental ill-health, and evaluate the study types and outcome measures used to evaluate them;

2. To conduct a systematic review of this evidence

**Methods:**

We searched the Cochrane Central Register of Controlled Trials (CENTRAL), CINAHL, DARE, Embase, MEDLINE, PsycINFO, and relevant government and non-government organisation websites for peer reviewed journal articles, including both qualitative and quantitative articles. Interventions were sorted into Categories and Types to manage heterogeneity.

**Results:**

We found 47 studies. Interventions were highly varied, and we created a taxonomy to understand this heterogeneity. Most studies were quasi-experimental trials (n=26; 55%) or qualitative studies (n=13; 27%) and only 8 RCTs (17%). Twelve were high quality (26%). Interventions were mostly found to have no effect on measured outcomes, though outcome measures may not have been best suited to expected domains of change.There was a broad range of outcome foci reflecting inconsistency in goals of interventions. No interventions were found to reduce the incidence of self-harm on the inpatient ward. One study suggests that inpatient safety planning may reduce readmission rates. Aggression-related outcomes for inpatient sensory modulation rooms were equivocal. Brief admissions with psychotherapy may reduce suicide attempt repetition and re-hospitalization, whereas brief admissions without psychotherapy may improve function but not re-hospitalization rates. Face-to-face psychoeducation for panic in the ED was associated with a reduction in ED presentation rates, but brochure-only psychoeducation may *increase* ED presentation rates.

**Conclusions:**

This review found little evidence to guide much of what clinicians do for people in crisis in hospital. There is a need to develop a framework for brief non-pharmacological interventions, address the quality and size of studies, and identify consistent outcome measures for non-pharmacological interventions. The data is insufficient to make clear recommendations for appropriate brief non-pharmacological interventions for people in crisis in Emergency Departments and Psychiatric Inpatient Units. Multiple promising interventions are available for further study, however there is a dearth of research and more rigorous testing is needed.

**Disclosure of Interest:**

None Declared

